# Surgically Treated Nonunion following Ischial Tuberosity Avulsion Fracture of a 14-Year-Old Athlete

**DOI:** 10.1155/2020/8531648

**Published:** 2020-06-12

**Authors:** Yuta Nakamatsu, Tomoaki Fukui, Keisuke Oe, Shinya Hayashi, Tomoyuki Matsumoto, Takehiko Matsushita, Ryosuke Kuroda, Takahiro Niikura

**Affiliations:** Department of Orthopaedic Surgery, Kobe University Graduate School of Medicine, Kobe, Japan, 7-5-2 Kusunokicho Chuo-ku, Kobe, Hyogo 650-0017, Japan

## Abstract

A 14-year-old girl experienced acute left buttock pain during a sprint. At the local hospital, she was diagnosed with an avulsion fracture of the left ischial tuberosity. She was kept for observation for about 10 months; however, the buttock pain persisted, and the bone fragments did not unite. She was referred to our hospital approximately 11 months after the injury. Plain radiography revealed an increased transposition of the bone fragment, from 12 mm immediately after the injury to 23 mm. Twelve months after the injury, she underwent osteosynthesis using two cannulated cancellous screws and three suture anchors. Following postoperative rehabilitation, the power in her left hamstring recovered, and she was able to run at full speed and returned to athletics 9 months after the surgery. The operative indications for avulsion fractures of the ischial tuberosity are unclear. Careful follow-up is required as the rate of nonunion after conservative treatment tends to be high. This needs to be identified in order to provide timely treatment that allows for early return to sport. Although she had significant chronic pain and muscle weakness, the surgery successfully treated the fracture, and her muscle power recovered, leading to her return to sports.

## 1. Introduction

Ischial tuberosity avulsion fractures occur due to mechanical loads, such as the contraction of the hamstrings, and are reported in adolescent athletes [1, 2]. They constitute about 30% of apophyseal avulsion fractures in the pelvis [2, 3]. Their incidence in males is reported to be twice as high as that in women [4]. They are often misdiagnosed as hamstring injuries because of the similarities in the mechanism of the injury and the sites of pain [5, 6]. As a result of delayed diagnosis or conservative treatment, many cases often develop nonunion, leading to chronic pain and leg-muscle weakness [7]. Surgical indications for the treatment for this fracture are unclear; thus, often leading to the development of nonunion following conservative treatment despite an accurate diagnosis. Although this occurs relatively commonly, few reports discuss the treatment for nonunion. This report presents a case of nonunion that developed after the surgical treatment of an ischial tuberosity avulsion fracture.

## 2. Case Presentation

The patient was a 14-year-old girl. We obtained informed consent from the patient and her parents for the publication of her data in this scientific study. She belonged to the athletic club of her junior high school. While sprinting in a relay race, she suddenly experienced left buttock pain and difficulty walking. The next day, she visited her nearby hospital and was diagnosed with an avulsion fracture of her left ischial tuberosity. The orthopedist continued to observe her without any treatment; however, 11 months after the injury, her pain had not improved, and the fracture did not achieve union. Therefore, she was referred to our department for further treatment.

At her first visit to our hospital, she was found to have local tenderness on her left buttock just above the ischial tuberosity. She could not sit for 10 minutes due to pain at the same point as the tenderness. Her hip and knee joints showed no restricted range of motion. There was no difference in the thigh and calf girth between both her legs. No abnormal neurologic finding was noted in both legs.

**Figure 1:**
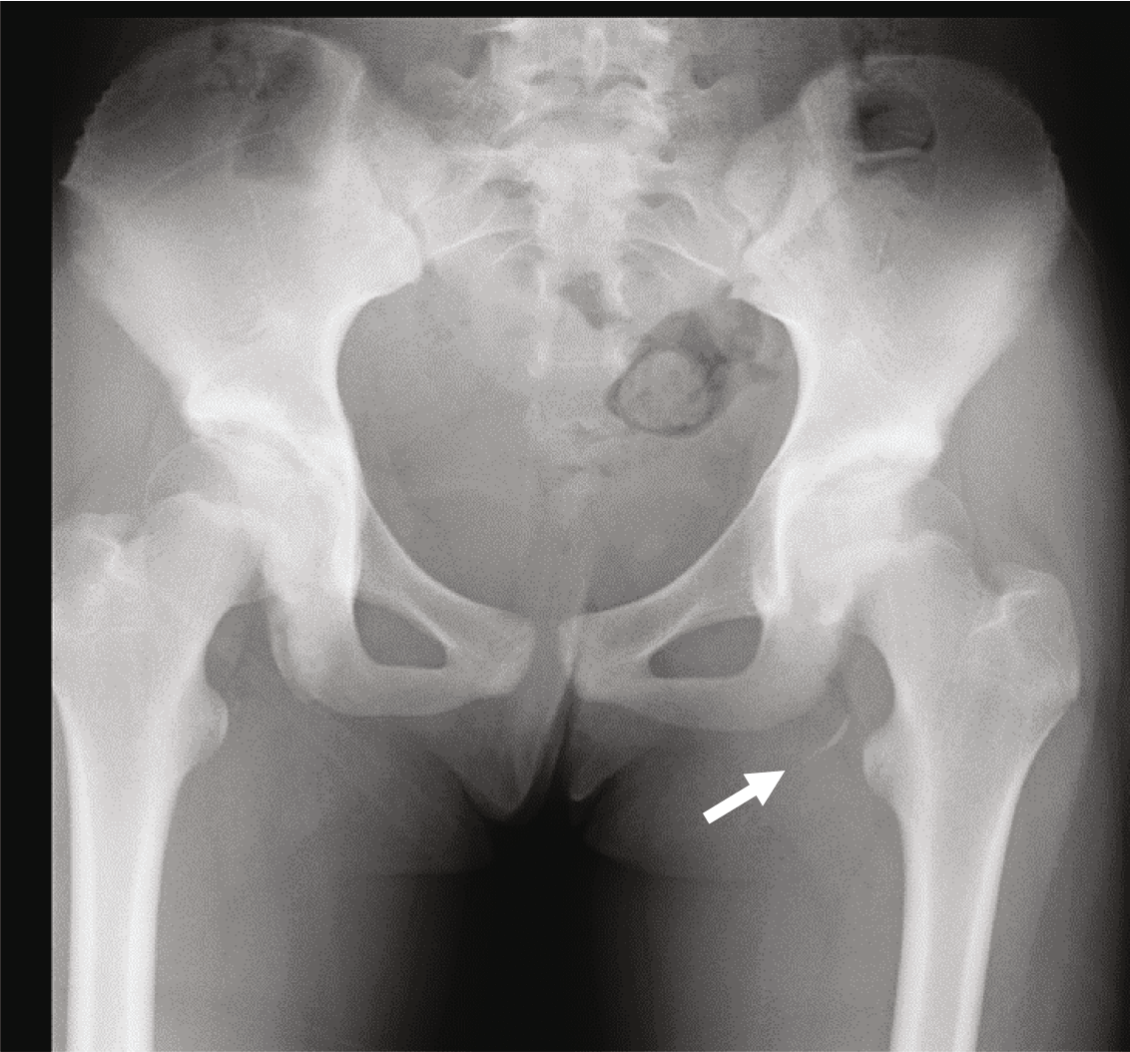
The X-ray image obtained on the day after the injury. A free bone fragment is found at her left ischial tubercle (arrow). Transposition of this fragment is about 12 mm.

**Figure 2:**
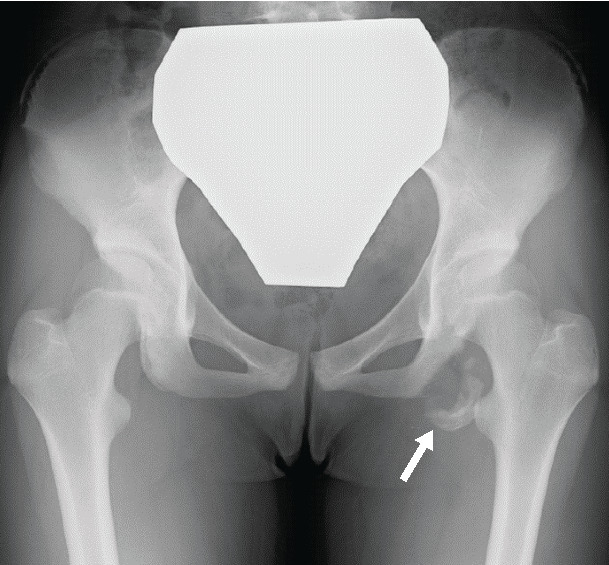
The X-ray image at the first visit to our hospital, 11 months after the injury. The bone fragment is enlarged, and the transposition is about 23 mm (arrow).

**Figure 3:**
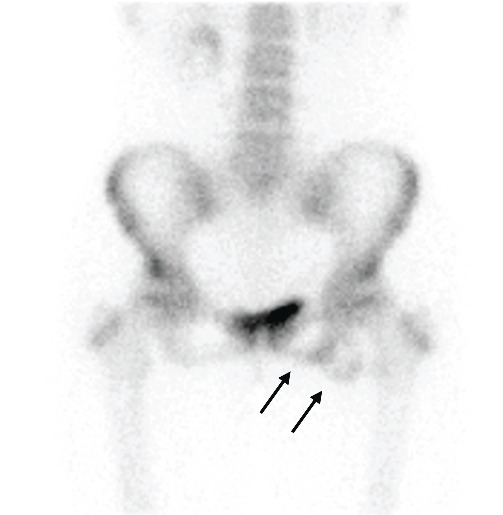
Bone scintigraphy performed before surgery. Intense uptake in the bone fragment and ischial tuberosity (arrows).

Radiographic images revealed the transposition of the bone fragment, which was 12 mm at the time of the injury (Figure 1) and had now increased to 23 mm, and the fragment had enlarged (Figure 2). Bone scintigraphy showed intense uptake in both sides of the nonunion site, ischial tuberosity, and bone fragment (Figure 3).

**Figure 4:**
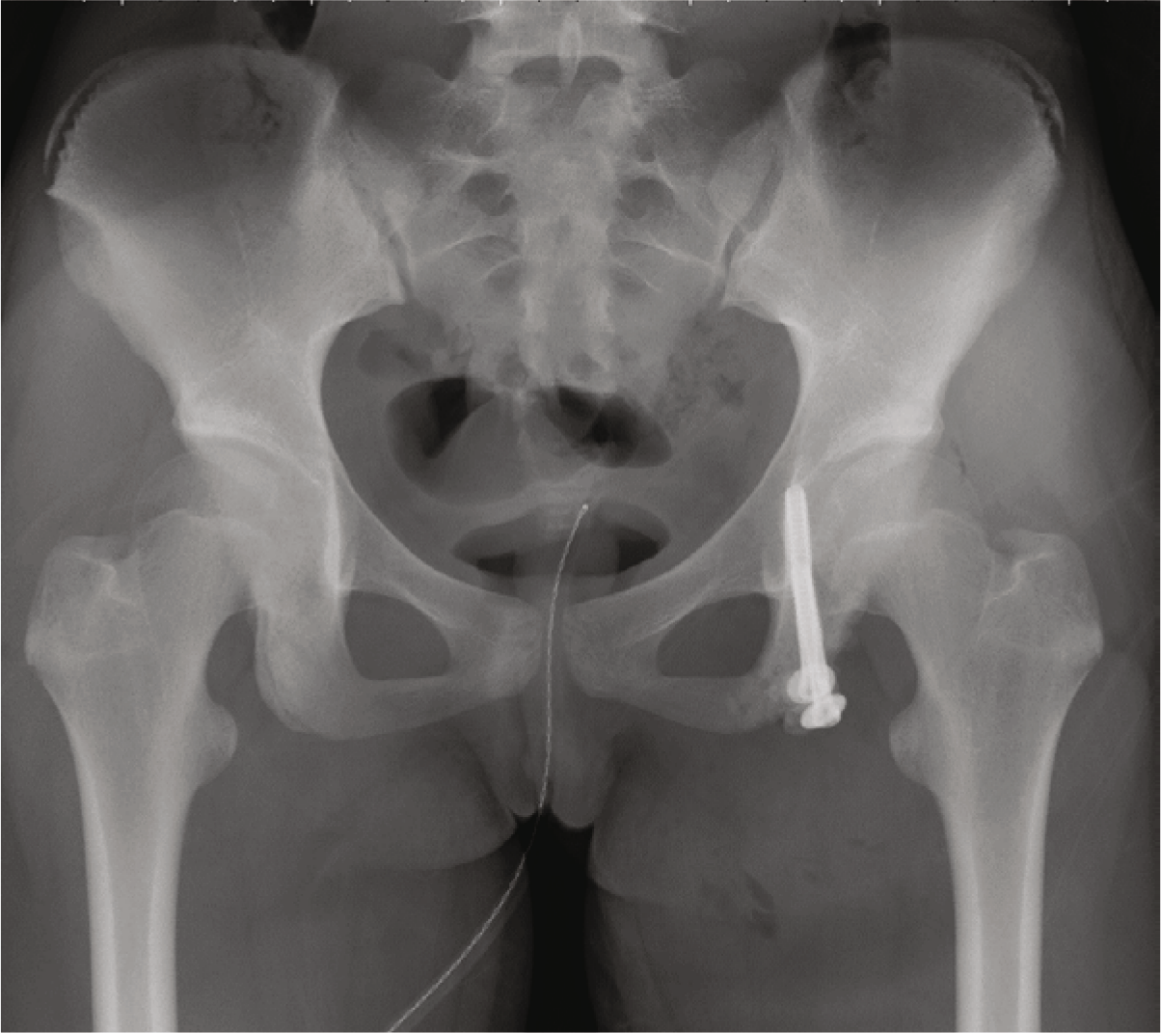
The X-ray image immediately after surgery. Two screws were inserted into her pelvic medulla in the direction to prevent them from penetrating the hip joint and to ensure the sufficient length.

She was diagnosed with nonunion following the avulsion fracture of her left ischial tuberosity, and surgical treatment was performed one year after the injury. The surgery was performed in the prone position under general anesthesia. An incision of about 10 cm was made along the gluteal crease. After lifting the gluteus maximus, the nonunion site was exposed. The unstable bone fragment was connected to the sciatic bone with capsule-like fibrous tissue, from which about 1 ml of bloody synovial fluid was withdrawn by a puncture. After removing the tissue around the bone fragment, it was fixed with two 6.5 mm-diameter cannulated cancellous screws. Then, the hamstring tendon was fixed to the ischial tuberosity with three 2.3 mm-diameter suture anchors (Figure 4).

**Figure 5:**
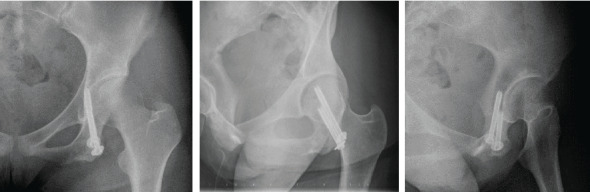
X-ray images 3 years after surgery. The bone fragment is firmly fused to her ischium.

From postoperative day 1, range of motion exercises of the hip and knee joints were allowed. During the first 4 weeks, any weight bearing on her left leg was prohibited. Thereafter, one-third partial weight bearing (PWB) was allowed, and the load was raised every 2 weeks in the following increments: 1/2 PWB, 2/3 PWB, and full weight-bearing. Jogging was started 3 months after the surgery when the radiographical bony union was recognized. We measured her hamstring strength before and after surgery in 90 degrees of knee flexion (Table 1) using a handheld dynamometer. Her hamstrings strength in 90 degrees of knee flexion on the affected side had improved to over 97% of that on the unaffected side 6 months after the surgery, and a strength of more than 85% was maintained even after the frequency of rehabilitation decreased. Nine months after the surgery, she could run at full speed and had returned to a competitive level of athletics. At the most recent follow-up, 3 years after the surgery, she had continued athletic activity in high school, without any pain (Figure 5).

## 3. Discussion

We reported a case of nonunion following an avulsion fracture of the ischial tuberosity, in which bony union was treated by fixation using screws and suture anchors, leading to muscle-power recovery.

The indications for surgically treating this fracture are not definite; however, developing a nonunion after nonsurgical treatment is possible. In a report by Ferlic et al., half of the patients with a displacement >15 mm had nonunion after conservative treatment; thus, he stated that surgery should be considered in patients with a displacement >15 mm [1]. Meanwhile, several authors suggested that fractures with a displacement >20 mm should be treated surgically [2, 8]. Furthermore, Gidwani et al. developed an original algorithm, according to which fractures with a displacement >10 mm needed early surgical fixation [9]. Schoensee et al. described cases with a bone fragment remaining ununited after 2 months of conservative treatment, which required surgical treatment [4]. There seems to be no difference between the rate of returning to the preinjury sport level after early and delayed surgeries [2]. In the current case, the displacement at the time of injury was 12 mm; however, 11 months after the injury, it was increased to 23 mm. Not only could she not return to club activity, but she was also unable to remain seated for 50 minutes in class. Because of the result of the lengthy conservative treatment, she eventually opted for surgery. In the case of conservative treatment for this kind of fracture, careful follow-up should be performed due to the rate of nonunion. The patient's desire to return to sports may also contribute to the indications for surgery.

The rarity of this report lies in the surgical method for treating the ischial tuberosity avulsion fracture with nonunion. Other reported procedures are plate fixation [10], bone transplantation [6], repair of the hamstring tendon with suture anchors after fragment excision [5], and surgical fenestration [4, 11]. We applied cancellous screws and suture anchors to fix the bone fragment, which led to the bony union. Bone grafting was not performed because intense uptake was shown around the nonunion area in the bone scintigraphy. Since this high uptake indicates the bioactivity of nonunion [12], we determined that it is possible to achieve bone fusion by osteosynthesis without bone grafting.

To our knowledge, there are few detailed reports evaluating muscle-strength recovery after the treatment of nonunion in ischial tuberosity avulsion fractures. In the case series by Schoensee et al., 3 patients received surgical fenestration, and their hamstring strength improved sufficiently 6 to 13 weeks postoperatively [4]. The muscle recovery of our patient was comparatively slower; however, our case had a wider dislocation of the bone fragment compared to that of those cases. Recovery was delayed, possibly due to her preoperative muscle weakness associated with the dislocation and severe pain interfering with her daily life.

## 4. Conclusion

We surgically treated a patient with nonunion of an ischial tuberosity avulsion fracture using cancellous screws and suture anchors without bone grafting, which resulted in her successful return to sports at the same level prior to the injury. The muscle weakness had also improved due to surgery and rehabilitation.

## Figures and Tables

**Table 1:** 

	Hamstrings strength		
	Right (nm/kg)	Left (nm/kg)	L/R
Pre-operation	0.40	0.45	112.5%
3 months	0.62	0.33	53.2%
6 months	0.83	0.81	97.6%
9 months	0.78	0.67	85.9%
12 months	0.83	0.73	88.0%
15 months	0.75	0.67	89.3%
24 months	0.67	0.60	89.6%

Pre- and postoperative data of the right and left hamstring strength measured by a handheld dynamometer in 90 degrees of knee flexion. The score represents the torque-weight ratio.
